# Pulmonary Arterial Hypertension in Indonesia: Current Status and Local Application of International Guidelines

**DOI:** 10.5334/gh.944

**Published:** 2021-04-20

**Authors:** Lucia Kris Dinarti, Dyah Wulan Anggrahini, Oktavia Lilyasari, Bambang Budi Siswanto, Anggoro Budi Hartopo

**Affiliations:** 1 Department of Cardiology and Vascular Medicine, Faculty of Medicine, Public Health and Nursing, Universitas Gadjah Mada—Dr. Sardjito Hospital, Yogyakarta, ID; 2 Department Cardiology and Vascular Medicine, Faculty of Medicine, Universitas Indonesia, National Cardiovascular Center Harapan Kita Jakarta, ID

**Keywords:** Pulmonary arterial hypertension (PAH), Congenital heart disease (CHD), etiology, Indonesia, international guideline, lower middle-income region

## Abstract

Recent advances in the diagnosis and management of pulmonary arterial hypertension (PAH) have led to a significant improvement in the outcomes for patients with PAH. However, prompt and accurate diagnosis of PAH remains an unmet challenge due to lack of awareness and lack of meticulous data to profile the etiology and pathophysiology of this rare progressive disease, especially in low- and middle-income country. In Indonesia, the true prevalence and incidence of different subtypes of PAH in general population is still unknown. The Congenital HeARt Disease in adult and Pulmonary Hypertension (COHARD-PH) registry was the first single-center prospective registry in Indonesia, which indicated that almost 80% of adult patients with congenital heart disease (CHD) had experienced PAH and even Eisenmenger syndrome due to delayed diagnosis. Screening for early detection of asymptomatic CHD in children is yet to be systematically established in Indonesia, leading to undiagnosed and uncorrected CHD in adulthood. There are no specific national guidelines focusing on diagnostic workup and treatment of PAH in Indonesia. Furthermore, the lack of adequate diagnostic facilities, limited treatment availability, and limited drug coverage under the National Health Insurance Scheme are key issues that remain unaddressed. This review focuses on the diagnosis, treatment, and management of PAH associated with CHD in Indonesia as per international guidelines. We have proposed recommendations to effectively control and prevent PAH associated with CHD in Indonesia. The paper should be of interest to readers in the area of medical management and policy makers especially in low- and middle-income countries.

**Key Highlights:**

1. Pulmonary arterial hypertension (PAH) is a rare progressive subtype of pulmonary hypertension with poor overall prognosis and outcomes.
2. Prompt and accurate diagnosis of PAH remains an unmet challenge in low- and middle-income countries due to poor knowledge about the etiology and pathophysiology of this syndrome. Also, the symptoms and signs of early-stage PAH are usually nonspecific or undetectable in newborn and infants, thus presenting a challenge for physicians to establish early diagnoses of PAH.
3. The challenging factors in low- and middle-income countries, especially Indonesia archipelago are limitations of healthcare infrastructure, limited expertise, lack of awareness, lack of timely PAH screening strategies, poor antenatal care and unpredictable availability of PAH medications.
4. There are no specific national guidelines focusing on diagnostic workup and treatment of PAH in Indonesia. Under-utilization of treatment guidelines and lack of adequate diagnostic treatment facilities have resulted in sub-optimal management of PAH patients in Indonesia.
5. Adherence to international guidelines is an important aspect of PAH management in Indonesia. Updated disease and functional classifications of PAH as per international guidelines along with new research findings on prognostic factors can help in making better management decisions for PAH patients at different stages of the disease.

## Introduction

Congenital heart disease (CHD) is the most common type of birth anomaly diagnosed in newborns [1], afflicting approximately 0.8%–1.2% of live births globally [2]. More than half of adult patients with CHD endure the risks of cyanosis, stroke, arrhythmias, and vascular complications, particularly in delayed diagnosis cases [3,4]. Pulmonary arterial hypertension (PAH) is a complication of CHD characterized by elevated mean pulmonary arterial pressure (mPAP > 20 mmHg at rest) and pulmonary vascular resistance (PVR ≥ 3 Wood Units), leading to right heart failure and premature death [5,6,7,8]. Pulmonary arterial hypertension is a rare progressive subtype of pulmonary hypertension (PH) with poor overall prognosis and outcomes [6]. Based on the etiology, PAH is classified into several categories: (a) idiopathic PAH (IPAH), (b) heritable PAH (HPAH), and (c) PAH associated with other conditions—such as connective tissue disease (CTD), CHD, portal hypertension, human immunodeficiency virus (HIV) infection, and exposure to toxins [6]. The symptoms and signs of early-stage PAH usually are nonspecific or undetectable in newborn and infants, thus presenting a challenge for physicians to establish early diagnoses of CHD-associated PAH. In high-income countries, the prevalence of adult CHD continues to rise due to improved survival attributed to successful CHD screening and surgical and medical management in childhood [7]. However, in low- and middle-income countries, many patients go undetected until complications arise that require medical attention [7]. With the exception of a handful of recent registries from Africa and the Middle East, the epidemiology of PAH in the low-and middle-income countries is still largely unknown [9]. The incidence of PAH in the low- and middle-income countries might be higher than that reported in high-income countries because of the prevalence of conditions, such as infection in pregnant women and preterm newborn babies, poor antenatal care, poor delivery services, and limited general physicians and pediatricians. There were also myths and beliefs of unhealthy attitude of traditional medicine. In addition, the prevalence of HIV, hemolytic anemias, schistosomiasis, liver cirrhosis, and chronic viral hepatitis might contribute to the development of PAH, although it has not yet been elucidated due to low PAH screening system in those diseases [9]. The challenging factors in low- and middle-income countries, especially Indonesia archipelago, are limitations of healthcare infrastructure, limited expertise, unpredictable availability of medications, lack of awareness about heart disease in new born and infants among family members, and limited diagnostic tools such as portable Doppler echocardiography in remote area [9].

To facilitate early disease diagnosis and management, the World Health Organization (WHO) classifies PH, including PAH, into four functional classes (FCs) based on disease severity (Table 1) [10]. Patients with WHO FC IV PAH have more severe symptoms and poorer prognosis compared to those with WHO FC I. According to the United States National Institutes of Health (US-NIH) registry, the median survival of WHO FC IV PAH patients if untreated is only around six months, compared with 2.5 years in patients with WHO FC III PAH and six years in patients with WHO FC I/II PAH [11].

**Table 1:** Functional classification of PH according to World Health Organization. PH: Pulmonary hypertension. Adapted from: Rich S. Primary pulmonary hypertension: executive summary. Evian, France: World Health Organization, 1998 [10].

Functional Class	Symptom or Level of Disease

Class I	Patients with PH but without resultant limitation of physical activity. Ordinary physical activity does not cause undue dyspnea or fatigue, chest pain, or near-syncope.
Class II	Patients with PH resulting in slight limitation of physical activity. Patients are comfortable at rest. Ordinary physical activity causes undue dyspnea or fatigue, chest pain, or near-syncope.
Class III	Patients with PH resulting in marked limitation of physical activity. Patients are comfortable at rest. Less-than–ordinary activity causes undue dyspnea or fatigue, chest pain, or near-syncope.
Class IV	Patients with PH with the inability to carry out any physical activity without symptoms. They manifest signs of right heart failure. Dyspnea and/or fatigue may even be present at rest. Discomfort is increased by any physical activity.

In Indonesia, the true prevalence and incidence of different subtypes of PAH is still unknown. The Congenital HeARt Disease in adult and Pulmonary Hypertension (COHARD-PH) registry was the first single-center, hospital-based registry that described the demographics, clinical presentation, and hemodynamic data of CHD-related PAH in Indonesia [7]. In addition, early screening for heart abnormalities in children has not yet been systematically established in Indonesia [7]. This situation affects the number of undiagnosed and uncorrected CHD in adulthood and also the outcomes of corrective management strategies in late finding PAH cases. Given the estimated cases of PAH is high based on that situation, there is a dire need for continued research to establish preventive strategies, timely screening, accurate assessment of disease severity, and timely management in Indonesia. Currently, there is a dearth of data on the management of PAH in Indonesia. This review article will focus on the diagnosis and management of PAH in Indonesia as per the established international guidelines.

## Diagnosis of PAH in Indonesia and Guideline Implementation

**Figure 1:**
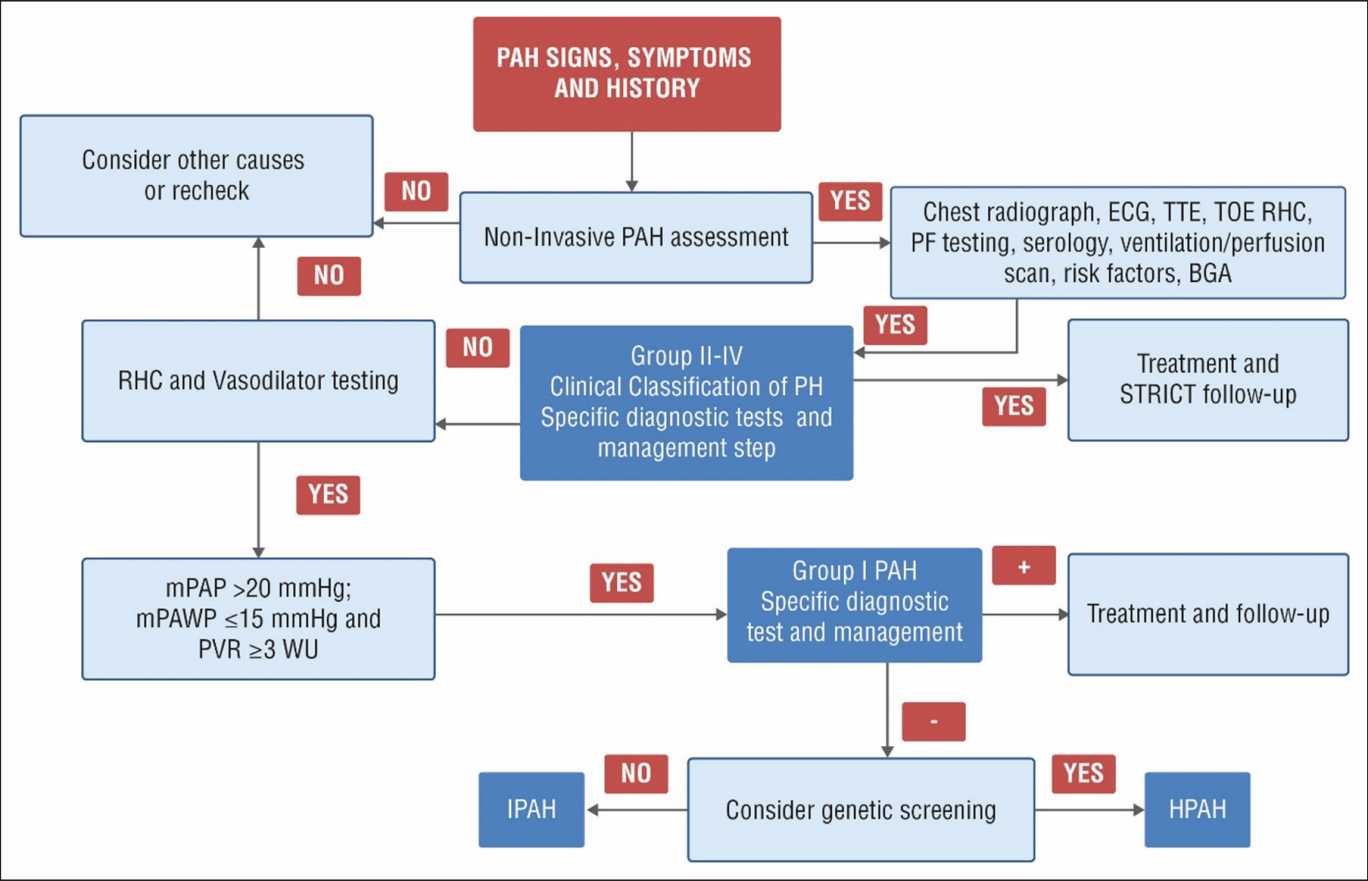
Diagnostic algorithm and different tests recommended for pulmonary arterial hypertension according to 2015 ESC/ERS guidelines and updated clinical classification of pulmonary hypertension. Adapted from: Simonneau G et al. [8], Galiè N et al. [12], and Desai AA et al. [13]. PH: Pulmonary hypertension; PAH: Pulmonary arterial hypertension; mPAP: Mean pulmonary arterial pressure; mPAWP: Pulmonary artery wedge pressure; PVR: Pulmonary vascular resistance; WU: Woods unit; IPAH: Idiopathic pulmonary arterial hypertension; HPAH: Heritable pulmonary arterial hypertension; ECG: Electrocardiogram; TTE: Transthoracic echocardiography; TOE: Transesophageal echocardiography; RHC: Right heart catheterization; PF: Pulmonary function; BGA: Blood gas analysis.

There are no specific national guidelines for the accurate diagnosis and management of PAH in Indonesia. Effective disease management strategies rely solely on the application of international guidelines. The 2015 European Society of Cardiology (ESC)/European Respiratory Society (ERS) guidelines strongly recommend regular assessment of PAH patients in the expert PH centers [12]. Detailed prognostic evaluation and risk assessment can provide information on comorbidities and disease complications associated with PAH. Figure 1 illustrates the diagnostic algorithm and clinical tests required for a comprehensive evaluation of PAH as per 2015 ESC/ERS guidelines and updated clinical classification of PH [8,12,13]. Patients with PAH usually have mild-to–moderate reduction in lung volume that can be investigated using pulmonary function tests [12]. Vasoreactivity testing is recommended in patients with IPAH, HPAH, and drug use-related PAH [12]. For CTD-related PAH, antinuclear antibody test and high-resolution computed tomography provide useful information for diagnosis [12,13].

A ventilation/perfusion scan (V/Q) is an important test for differentiating IPAH from chronic thromboembolic PH [14]. Underutilization of V/Q scans during the screening of PH patients might lead to misdiagnosis of PAH [14].

### Prognostic Risk Evaluation in PAH Patients as per International Guidelines

Table 2 lists the tests recommended by ESC/ERS guidelines for comprehensive prognostic risk evaluation in PAH patients [12]. These include simple clinical tests, exercise testing, hemodynamic measurement, and biomarker tests. Imaging studies (e.g. echocardiography and cardiac magnetic resonance imaging) are essential in constructing a prognosis as symptoms of early-stage PAH are non-specific and for managing signs of complications [7,12]. Echocardiography is used for the assessment of right ventricular (RV) structure and function, indexed right atrial area (RA_area_), and pericardial effusion [15]. The six-minute walk test (6MWT) and cardiopulmonary exercise testing (CPET) are two standard tests for measuring exercise capacity [12,15]. The brain natriuretic peptide [BNP]/N-terminal fragment of pro-BNP [NT-pro-BNP] biomarker is an essential prognostic indicator for heart failure and recommended as a test for PAH risk stratification [12,15]. Right heart catheterization is the diagnostic gold-standard test for reliably confirming PAH [16,17]. Hemodynamics assessed by right heart catheterization (RHC) provides important prognostic information such as cardiac index (CI), mixed venous oxygen saturation (SvO_2_), PAP, pulmonary artery wedge pressure (PAWP), RAP, and right ventricular pressure (RVP) [12,16,17].

**Table 2:** Determinants of PAH diseases severity and prognosis. Adapted from: Galiè N, et al. [12] BNP/NT-pro-BNP: Brain natriuretic peptide/N-terminal fragment of pro-BNP; CI: Cardiac index; CPET: Cardiopulmonary exercise testing; FC: Functional class; 6MWT: 6-minute walking test; RAP: Right atrial pressure; RV: Right ventricular; WHO: World Health Organization; RA: Right atrial.

Determinants of Prognosis	Details	Low Risk	Medium Risk	High Risk

Clinical signs of RV failure evidence		Absent	Absent	Present
FC [12,16]		Classes I, II	Class III	Class IV
Progression of symptoms [12,16]		No	Slow	Rapid
Exercise testing [12]	6MWT	>440m	165–440 m	<165 m
	CPET	Peak oxygen consumption > 15 mL/min/kg	Peak oxygen consumption 11–15 mL/min/kg	Peak oxygen consumption < 11 mL/min/kg
Clinical test [12,15]	Echocardiographic findings	No pericardial effusion	No or minimal pericardial effusion	Pericardial effusion
		RA area <18 cm^2^	RA area 18–26 cm^2^	RA area > 26 cm^2^
	Hemodynamics	RAP < 8mmHg CI ≥ 2.5L/min/m^2^	RAP 8–14 mmHg CI 2–2.4L/min/m^2^	RAP > 14 mmHg CI ≤ 2.0 L/min/m^2^
Biomarker test [12,15]	BNP/NT-pro-BNP plasma levels	Normal BNP < 50 ng/L, NT-pro-BNP < 300ng/L	Elevated BNP: 50–300 ng/L, NT-pro-BNP: 300–1400 ng/L	Very elevated BNP > 300 ng/L, NT-pro-BNP > 1400 ng/L

### Diagnostic Strategy Adopted in Indonesia as per International Guidelines

The diagnostic strategy in the COHARD-PH registry provides information on international guideline implementation in Indonesia. In the registry, patients (n = 1012 aged 18 years or older) were interviewed and underwent physical examination, electrocardiogram (ECG) examination, 6MWT, and a chest X-ray examination [7]. Oxygen saturation assessment during the 6MWT test determined the degree of hypoxemia [7,18]. To confirm the diagnosis of CHD, transthoracic echocardiography (TTE) was performed [7]. Through TTE, the probability of PH was assessed based on the 2015 ESC/ERS guideline recommendations [7,12]. Transesophageal echocardiography (TOE) was performed in CHD patients with confirmed atrial septal defect (ASD) and ventricular septal defect (VSD) after the TTE examination [7]. Right heart catheterization was later performed in patients for diagnosing PAH and measuring hemodynamics [7]. Image acquisition, validation, and confirmation were as per the European Association of Echocardiography (EAE) and American Society of Echocardiography (ASE) guidelines [7].

**Figure 2:**
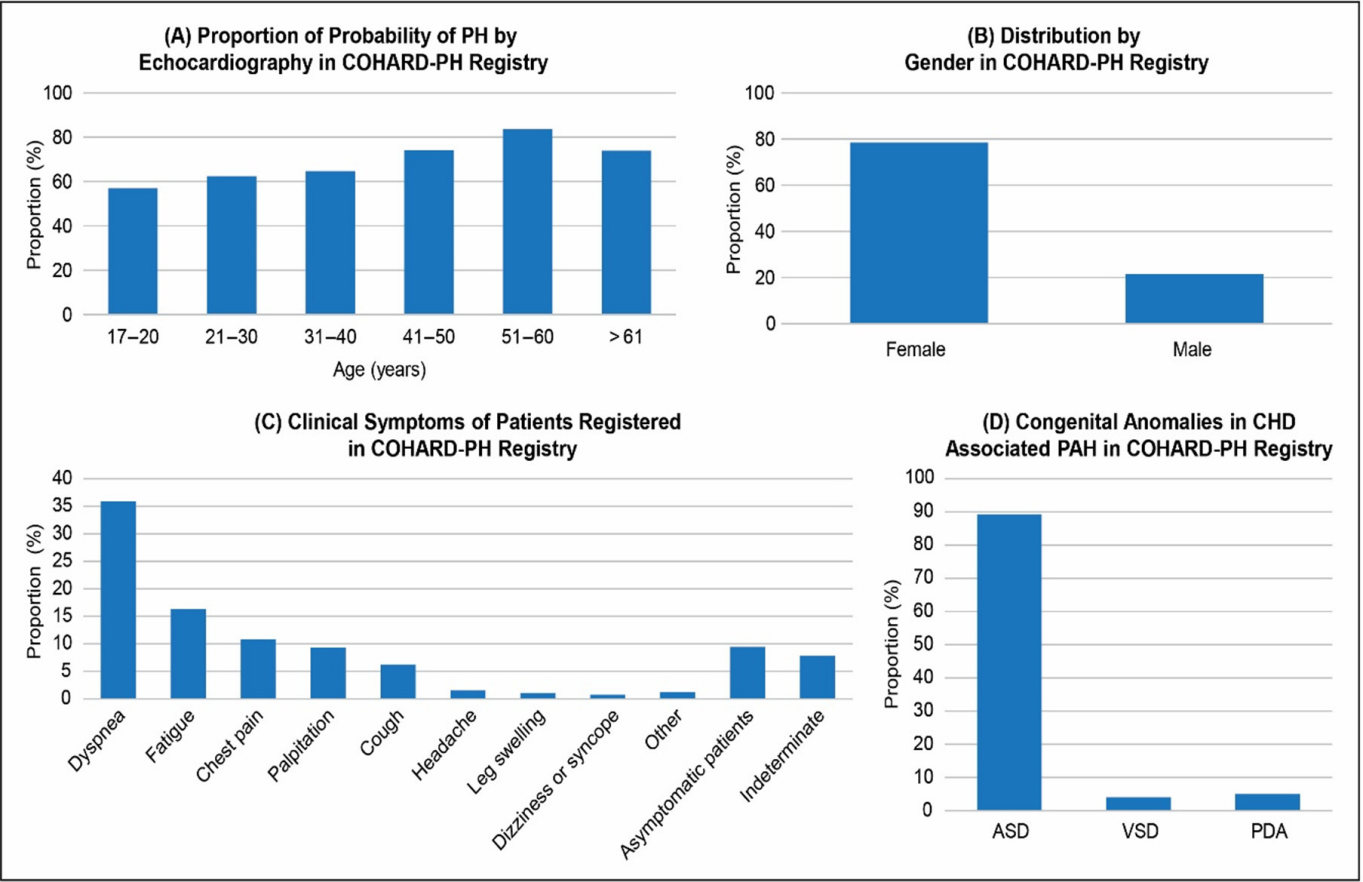
Results from COHARD-PH registry (2012–2019) in Indonesia in all registered patients (n = 1012) [7]. **a)** Proportion of probability of PH by echocardiography; **b)** Distribution by gender; **c)** Clinical symptoms; **d)** Congenital anomalies in CHD-associated PAH. CHD: Congenital heart disease; PAH: Pulmonary arterial hypertension; COHARD-PH: Congenital heart disease in adult and pulmonary hypertension; ASD: Atrial septal defect; VSD: Ventricle septal defect; PDA: Patent ductus arteriosus.

The application of the ESC Guidelines on the diagnosis of PH in CHD was shown in COHARD-PH registry [7]. The echocardiographic data showed that 77.1% of the enrolled patients had an increased probability of developing PH [7]. The RHC results confirmed that 66.9% of patients had developed PAH [7]. About 72.7% of patients had been asymptomatic for greater than two decades [7]. At the time of diagnosis, 43% of patients belonged to FC II group and 42% to FC I group [7]. The highest proportion of CHD-related PAH belonged to the age group of 51–60 years (Figure 2a), largely due to delayed prognosis [7]. The most common complaints (Figure 2c) reported were dyspnea on effort (35.9%), fatigue (16.3%), and chest pain/discomfort (10.8%) [7]. Atrial septal defect (ASD) was the most common (Figure 2d) congenital heart defect (89.3%) among PAH patients (aged ≥18 years), followed by patent ductus arteriosus (PDA) (5.1%) [7]. The signs of Eisenmenger syndrome were encountered in 18.7% of patients [7].

Among registered patients who underwent RHC (n = 614), those with CHD-related PAH were predominantly young adult females (Figure 2b), who accounted for 81.8% of cases (n = 336) [7]. Patients with CHD-related PAH had a significantly older age at first diagnosis (p < 0.001), lower 6-min walking distance (p < 0.001), worse WHO functional class (WHO FC III–IV: 14.2% vs. 5.0%, p < 0.001), and higher biomarker NT-pro-BNP levels (median: 774.0 vs. 121.5 pg/mL, p < 0.001) compared to CHD patients without PAH (Table 3) [7].

**Table 3:** Comparison of clinical parameters between CHD-related PAH and CHD without PAH after diagnosis by RHC (n = 614). Adapted from Dinarti LK, et al. [7]. ASD: Atrial septal defect; BNP/NT-pro-BNP: Brain natriuretic peptide/N-terminal fragment of pro-BNP; COHARD-PH: Congenital heart disease in adult and pulmonary hypertension; FC: Functional classification; IQR: Interquartile range; mPAP: Mean pulmonary arterial pressure; mRAP: Mean right atrial pressure; PDA: Patent ductus arteriosus; RHC: Right heart catheterization; VSD: Ventricle septal defect; WHO: World Health Organization.

Characteristics of CHD Patients Based on PAH Diagnosis By RHC (N = 614)		

	CHD-related PAH (n = 411)	CHD without PAH (n = 203)

Age at enrollment (years) (mean ± SD)	36.4 ± 12.9	32.2 ± 12.0
Gender (n, %)	Males: 75,18.2	Males: 42, 20.7
	Females: 336, 81.8	Females:161, 79.3
Congenital abnormalities (n, %)	ASD: 367, 89.3	ASD: 166, 81.8
	VSD:17, 4.1	VSD: 26,12.8
	PDA: 21, 5.1	PDA: 10, 4.9
6-minute walk distance (meters) (mean ± SD)	336.3 ± 99.7	393.9 ± 82.1
WHO Functional class (n, %)	FC 1: 136, 34.0	FC 1: 122, 60.4
	FC II: 207, 51.8	FC II: 70, 34.7
	FC III–IV: 57, 14.2	FC III–IV: 10, 5.0
NT-pro-BNP (pg/mL)(median [IQR])	774.0 (242.8–2022.3)	121.5 (57.1–218.1)

Most CHD-related PAH patients (with uncorrected ASD) in Indonesia belonged to the intermediate-risk group, according to the 6MWT results [7,19]. In Indonesia, pregnant women with PH related to uncorrected CHD have a high risk of maternal mortality (12.5%), which was higher than the previous study by Hartopo et al. (10.7%) [20]. This study indicated that 64.3% patients were diagnosed with CHD at the time of pregnancy [20]. It was observed during the study that no antenatal care for CHD condition was done to the patients [20]. Additionally, most pregnant patients who presented to the hospital were already in their third trimester of pregnancy.

## Treatment Strategy According to 2015 ESC/ERS Guidelines for Management of PAH

**Figure 3:**
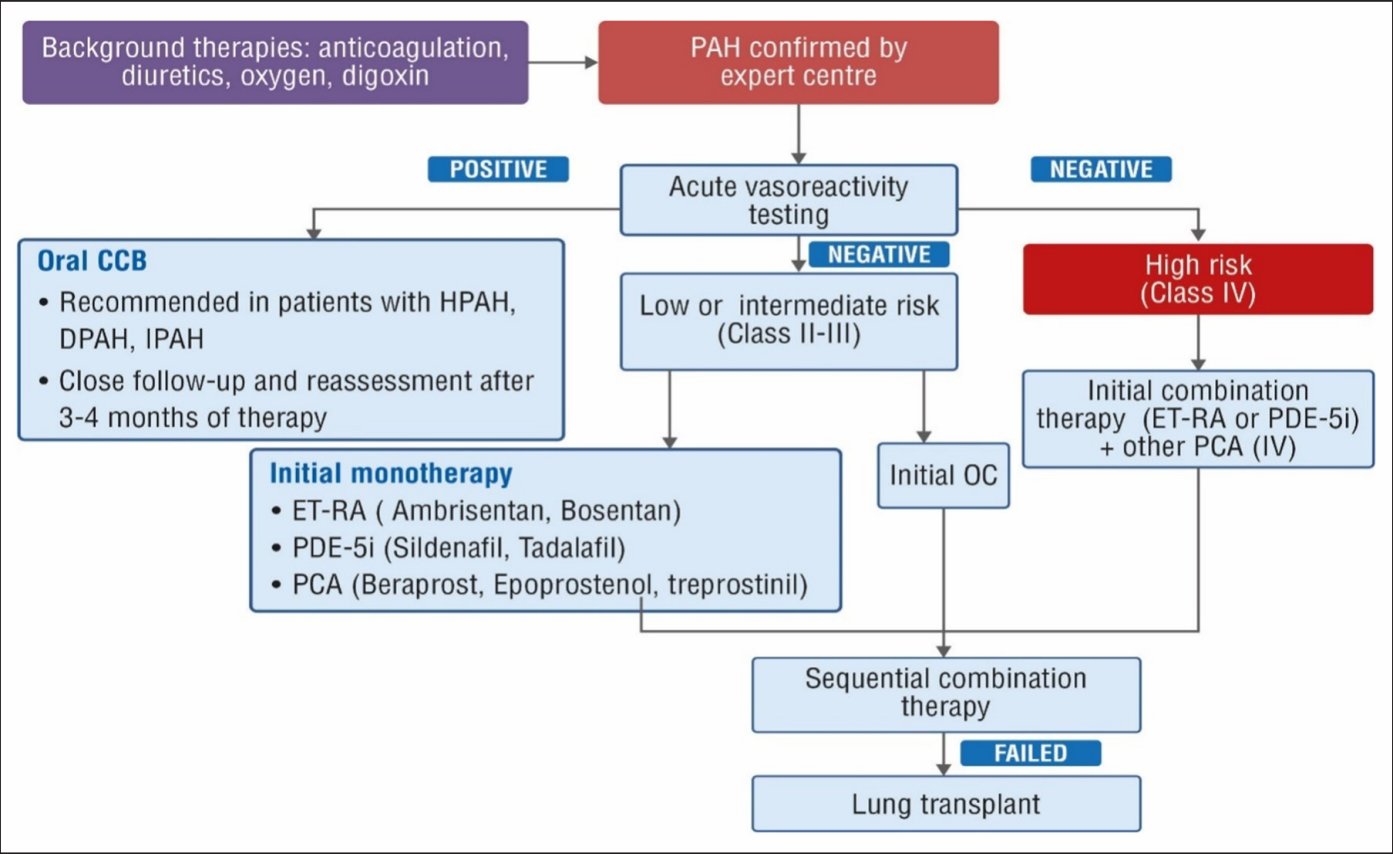
Treatment algorithm for pulmonary arterial hypertension according to 2015 ESC/ERS guidelines. Adapted from: Galiè N, et al. [12], McLaughlin VV et al. [21], and Taichman DB et al. [23] PAH: Pulmonary arterial hypertension; CCB: Calcium-channel blocker; IPAH: Idiopathic pulmonary arterial hypertension; HPAH: Heritable pulmonary arterial hypertension; DPAH: Drug-induced pulmonary arterial hypertension; ET-RA: Endothelin receptor antagonist; PDE-5i: Phosphodiesterase type 5 inhibitor; PCA: Prostacyclin analog; OC: Oral combination.

Optimal therapy for a patient with PAH is highly individualized and depends on the disease severity, route of administration, side effects, treatment goals, and clinician preferences [12]. As per 2015 ESC/ERS guidelines, general/basic therapies include warfarin anticoagulants, diuretics (for management of RH failure), oxygen (to reduce PVR), and digoxin (in patients with atrial arrhythmias) [12,21]. Patients with PAH due to conditions other than IPAH and HPAH have a very low rate of long-term responsiveness to oral calcium-channel blockers (CCBs) [21]. A treatment algorithm for the therapy of PAH is depicted in Figure 3. For patients who are acute vasodilator-tested negative and considered to have a lower or intermediate PAH risk based on clinical assessment (Table 2), therapy with endothelin receptor antagonists (ET-RA) or phosphodiesterase type 5 inhibitors (PDE-5i) would be the recommended first-line therapy [21]. In a quasi-experimental study conducted at Dr. Sardjito Hospital Yogyakarta, Indonesia, sildenafil (an oral PDE-5i), when used in PAH patients with uncorrected ASD (WHO FCs II–III), significantly reduced PAH symptoms and resulted in an overall improvement in health-related quality of life [22]. For patients who are considered high-risk based on clinical assessment, combination therapy (ET-RA or PDE-5i with intravenous [IV] prostacyclin [epoprostenol or treprostinil]) would be the first-line therapy [12,21,23]. Sequential combination therapy is advised if all the above therapies fail to provide any treatment benefit [12].

Increased mPAP in PAH patients with uncorrected ASD is attributed to increased endothelin-1 levels [24,25] According to a single-center retrospective study performed in Indonesia, a significant positive and moderate correlation (r = 0.423 and p = 0.004) exists between vasoconstrictor endothelin-1 level and mPAP [25]. Therefore, in early-phase PAH (mild-to–moderate PAH), ET-RA is important to reduce mPAP [15,24,25]. On the contrary, prostacyclin analogs and PDE-5i have their most pronounced impact in late-phase PAH [25]. The latter could be because vasodilator substances (prostacyclin and nitric oxide) are increased in mild-to–moderate PAH, but decreased in severe PAH [25].

## Management of PAH in Indonesia

Whilst there are eleven drugs used for PAH treatment, only two drug classes are commonly used in Indonesia. Prostacyclin analogs (beraprost and iloprost) and oral PDE-5i (sildenafil) are the currently available PAH drug classes in Indonesia [26,27]. ET-RAs (bosentan, ambrisentan and macitentan) approved for the treatment of high-risk PAH patients (WHO FCs III–IV) are not accessible in Indonesia [15,26]. In 2014, the Indonesian government introduced a new health insurance scheme, known as Jaminan Kesehatan Nasional–Kartu Indonesia Sehat (JKN-KIS), managed by Indonesia’s National Healthcare Security Agency (BPJS-Kesehatan) [28]. The National Formulary (NF) is a reimbursement policy and provides a list of medications covered by BPJS-Kesehatan. There is limited drug coverage for PAH under this scheme, i.e. oral beraprost and sildenafil; and the most recent approved treatment was inhaled iloprost [26]. In an economic evaluation study published by Lilyasari et al., generic sildenafil was found to be more cost-effective for the treatment of FCs II and III PAH patients in Indonesia as compared to beraprost [26]. Despite being more expensive than beraprost, sildenafil yielded 1–3 additional life-years compared to beraprost [26]. Also, sildenafil is a cost-effective therapy for PAH with a low price and a net increase in quality-adjusted life years compared to other approved therapies (bosentan, treprostinil, epoprostenol, inhaled iloprost, sitaxentan, and ambrisentan) [26]. Furthermore, sildenafil is well-tolerated among children with IPAH and in PAH associated with CHD [29]. The European Medicines Evaluation Agency (EMEA) recommends the use of sildenafil in pediatric PAH patients (aged 1–17 years) based on body weight with a maximum daily dosage of 10 mg thrice daily (weight < 20 kg) or 20 mg thrice daily (weight > 20 kg) [30]. Although the published literature has generally suggested favorable outcomes in the pediatric PAH subgroup, caution must be exercised regarding long-term use at higher doses in this group of patients.

The physical activity and supervised cardiopulmonary rehabilitation program is recommended as part of PAH management program as it has shown to improve functional capacity and quality of life (QoL) in patients [12]. In a randomized controlled study among CHD-related PAH patients in Indonesia, the combination of hospital and home-based physical exercise program added to PAH-targeted therapy, mostly sildenafil, improved functional capacity and QoL in patients [31].

## Addressing Challenges and Unmet Needs in PAH Management in Indonesia

Recent advances in the diagnosis and management of PAH have resulted in a significant improvement in outcomes for patients with PAH in Indonesia. However, prompt and accurate diagnosis of PAH still remains a challenge due to poor knowledge about the etiology and pathophysiology of this rare abnormality. Under-utilization of treatment guidelines and lack of diagnostic treatment facilities (lung V/Q scan, RHC, cardiac computed tomography, and cardiac magnetic resonance imaging) result in sub-optimal treatment of PAH patients in Indonesia [32]. Cost burden and limited drug coverage under the National Health Insurance are key issues that must be addressed by the government to improve PAH outcomes in Indonesia [29,32,33]. Annual screening for elementary school children and pregnant women, by cardiac auscultation and 12-lead electrocardiography, can help improve PAH outcomes in Indonesia since it can detect asymptomatic CHD [34]. A recent study published by Dinarti LK, et al., used these methods for screening CHD among Indonesian elementary school students [34]. The study indicated the cardiac abnormality prevalence of 2.9 per-1000 school-age children [34]. Implementing these simple, reliable CHD screening methods in school-age children can provide early referral and appropriate management and reduce mortality associated with undetected CHD later in adulthood.

## Recommendations and Future Directions

- Firstly, efforts to diagnose and treat PAH patients in Indonesia need to be scaled up further. In addition to registries at national referral centers, there is a dire need for meticulous data collection across different hospitals in Indonesia to estimate the true prevalence of PAH. Such a nationwide registry may hold great prominence in the future for designing and promoting standards of care in PAH. In Indonesia, nationwide screenings in the prenatal and postnatal periods have not yet been implemented [34].
- Secondly, PAH care is still centralized in Indonesia, with patients being referred to tertiary health facilities for comprehensive evaluation and management which leads to later diagnosis. Owing to the nature of PAH, early detection is the key to successful management. It is important to heighten awareness among healthcare providers at all tiers of the healthcare system about the pathophysiology of this condition, prognostic and risk factors, and the pressing need for early detection and management. Specialized training programs on PAH for cardiologists, pediatricians, and other specialties can be beneficial. Pediatricians are uniquely placed to pre-emptively assess PAH risk in children with CHD. Setting up PAH specialty clinics across Indonesia can also help improve access to expert care. The establishment of dedicated innovation research centers for PAH treatment and management will also help improve patient outcomes in Indonesia.
- Thirdly, more drug variants for PAH should also be made available in the NF for effective PAH management in Indonesia. Comparative analysis of costs associated with adverse effects in patients receiving upfront combinational therapy versus monotherapy should be evaluated. Also, supportive care (physical activity and supervised cardiopulmonary rehabilitation program) should not be overlooked for PAH patients as it can increase exercise capacity and improve QoL [12,31].
- Lastly, adherence to international guidelines is an important aspect of PAH management in Indonesia. Updated disease and functional classifications of PAH as per international guidelines [8,10,12,13], along with new research findings on prognostic factors could offer key support for making effective therapy and management decisions for PAH patients at different stages of the disease. However, it is important to reduce regional variations in PAH management and promote the adoption of best practices across Indonesia.

## Conclusion

Pulmonary arterial hypertension (PAH) is a rare but debilitating medical condition. There are several challenges and unmet needs relating to PAH diagnosis and treatment in Indonesia. Effective management of PAH can be facilitated by developing effective screening strategies, promoting early diagnosis and by applying international guidelines. To increase understanding of the prevalence and incidence of PAH and its subtypes, future efforts should focus on building a nationwide registry and investing in PAH clinical research.
